# Molecular Cytogenetic Identification of a New Wheat-Rye 6R Addition Line and Physical Localization of Its Powdery Mildew Resistance Gene

**DOI:** 10.3389/fpls.2022.889494

**Published:** 2022-05-12

**Authors:** Guohao Han, Hanwen Yan, Jing Wang, Lijun Cao, Shiyu Liu, Xiuquan Li, Yilin Zhou, Jieru Fan, Lihui Li, Diaoguo An

**Affiliations:** ^1^Center for Agricultural Resources Research, Institute of Genetics and Developmental Biology, Chinese Academy of Sciences, Shijiazhuang, China; ^2^National Key Facility for Crop Gene Resources and Genetic Improvement, Institute of Crop Science, Chinese Academy of Agricultural Sciences, Beijing, China; ^3^State Key Laboratory for Biology of Plant Diseases and Insect Pests, Institute of Plant Protection, Chinese Academy of Agricultural Sciences, Beijing, China; ^4^Innovative Academy of Seed Design, Chinese Academy of Sciences, Beijing, China

**Keywords:** powdery mildew, *Secale cereale*, *Triticum aestivum*, chromosome-specific marker, 6R

## Abstract

Rye (*Secale cereale* L.), a naturally cross-pollinating relative of wheat, is a tertiary gene donor and of substantial value in wheat improvement. Wheat powdery mildew is caused by *Blumeria graminis* f. sp. *tritici* (*Bgt*), which seriously affects yield and quality worldwide. Identifying and transferring new, effective resistance genes against powdery mildew from rye is important for wheat breeding. The current study developed a wheat-rye line YT2 resistant to powdery mildew by crossing, backcrossing, and self-pollination for multiple generations between octoploid triticale 09R2-100 and common wheat cultivar Shixin 616. YT2 was confirmed to be a 6R disomic addition and T1RS⋅1BL translocation line by genomic *in situ* hybridization (GISH), multicolor fluorescence *in situ* hybridization (mc-FISH), multicolor-GISH (mc-GISH), and molecular marker analyses. Disease responses to different *Bgt* isolates and genetic analysis showed that the powdery mildew resistance gene of YT2 was derived from the rye chromosome 6R of 09R2-100, which differed from the previously reported *Pm* genes from rye including *Pm20* on 6RL. Resistance phenotype of different translocation lines and deletion lines derived from YT2 combined with newly developed 6RL-specific markers analysis suggested that the powdery mildew resistance gene of YT2 was localized to the region in chromosome 6RL: 890.09–967.51 Mb and flanked by markers *XM189* and *X4M19*, corresponding to the reference genome of Weining rye. Therefore, YT2 could be used as a promising bridging parent for wheat disease resistance improvement.

## Introduction

Common wheat (*Triticum aestivum* L.) is one of the most important grain crops worldwide ensuring national food security. Powdery mildew, caused by *Blumeria graminis* (DC.) Speer f. sp. *tritici* emend. É. J. Marchal (*Bgt*), can significantly decrease wheat yield and quality worldwide by 5–40% and, in severe cases, up to 62% (Singh et al., 2016; Sun et al., 2018). Using host resistance is considered the most effective and sustainable way of controlling this disease, so a large number of powdery mildew (*Pm*) resistance genes/alleles continue to be explored, with more than a hundred *Pm* genes (*Pm1*–*Pm68*, noting that *Pm8* = *Pm17*, *Pm18* = *Pm1c*, *Pm22* = *Pm1e*, *Pm23* = *Pm4c*, and *Pm31* = *Pm21*) reported so far (McIntosh et al., 2020; He et al., 2021). However, most of the *Pm* genes cannot be directly applied in wheat breeding programs due to undesirable linkage drag. In addition, *Pm* genes are easily overcome after long periods of utilization in practical production by the highly variable pathogen (Mohler and Stadlmeier, 2019). A well-known example is the “boom-bust” of *Pm8*, which has caused continual severe epidemics in the main wheat production regions in China (He et al., 2011, 2015; An et al., 2019). Therefore, it is a continuously urgent need to explore and utilize sustainable resistance against powdery mildew in new gene resources.

For a long time, wheat breeding has mainly focused on the crossing between cultivars, leading to homogeneous genetic backgrounds and narrowed genetic diversity (Gupta et al., 2010). A growing number of studies have revealed the immense potential of mining useful genes from wheat relatives (Friebe et al., 1996; Colmer et al., 2006; Tester and Langridge, 2010; Chen et al., 2013; Hurni et al., 2015; Li H.H. et al., 2020; Li J.B. et al., 2020). Rye (*Secale cereale* L.) is a naturally cross-pollinating relative of common wheat, which possesses huge diversity for using as a gene donor in wheat improvement (Targońska et al., 2016; An et al., 2019). Many resistance genes on chromosome 6R of rye have been identified and transferred into wheat, including *Pm56* on 6RS from Qinling rye and *Pm20* on 6RL from Prolific rye for powdery mildew resistance (Friebe et al., 1994; Hao et al., 2018); *Yr83* on 6RL from T-701 triticale derivatives for stripe rust resistance (Li J.B. et al., 2020); *CreR* on 6RL from T-701 triticale derivatives for cereal cyst nematode (*Heterodera avenae* Woll.) resistance (Taylor et al., 1998); and *H25* on 6RL from Balbo rye for Hessian fly resistance (Friebe et al., 1991, 1996). Apart from these formally designated resistance genes, some wheat-rye 6R derivative lines have also exhibited resistance that was identified to be derived from rye. For instance, 6R from German White rye and 6RL from Kustro rye both conferred resistance to powdery mildew (Fu et al., 2014; An et al., 2015; Du et al., 2018). The chromosome 6R from Kriszta rye appeared to be resistant to stripe rust (Schneider et al., 2016). The recently published reference genome of Weining rye revealed that as many as 287 disease resistance-associated genes were predicted on chromosome 6R (Li et al., 2021). So, widening the variation of 6R to achieve its full potential for novel resistance gene resources is an ongoing and attractive prospect.

Molecular markers specific for alien chromosomes are powerful for easily detecting alien chromatin in the wheat background (Hechanova et al., 2018). To date, many types of molecular markers specific for the chromosome 6R were developed (Korzun et al., 1998; Stojalowski et al., 2009; Milczarski et al., 2011; Xu et al., 2012; Li et al., 2013, 2016; Qiu et al., 2016; Du et al., 2018; Han et al., 2020a; Li J.B. et al., 2020), which has accelerated gene mapping, improved the accuracy of germplasm selection, and promoted germplasm innovation. Nevertheless, the number of markers covering the entire chromosome 6R is still in large demand, especially for those with accurate physical location information and tightly linked to the targeted genes. Fortunately, the recent release of the high-quality rye reference genome sequence can greatly accelerate the development of rye-specific markers and the identification of useful genes (Li et al., 2021; Rabanus-Wallace et al., 2021).

Octoploid triticale (× *Triticosecale* Wittmack, 2n = 8x = 56, AABBDDRR) is synthesized artificially through distant hybridization between common wheat and rye. It possesses multiple resistance genes to wheat disease and can be more easily crossed with wheat than rye, thus can be used as an alternative source in wheat improvement (Oettler et al., 2005; Li et al., 2018). In the present study, after many years of selection of disease resistance and satisfactory agronomic traits, a stable wheat-rye 6R derivative line YT2 with high resistance to powdery mildew at the adult plant stage was obtained. To better understand YT2 and to facilitate the utilization of its powdery mildew resistance, this study reported the following: (1) its chromosome composition; (2) the assessment and genetic analysis of its powdery mildew resistance; (3) the development of chromosome arm 6RL-specific markers; (4) the physical localization of its powdery mildew resistance gene; and (5) the evaluation of its agronomic performance for breeding.

## Materials and Methods

### Plant Materials

A wheat-rye derivative line, designated as YT2, was produced by crossing octoploid triticale 09R2-100 (2n = 8x = 56, AABBDDRR) with wheat cultivar Shixin 616, with self-pollination for multiple generations. Wheat cultivar Mingxian 169 susceptible to all the *Bgt* isolates tested was used as a susceptible control for resistance assessment to powdery mildew at both seedling and adult stages. To evaluate the powdery mildew resistance of YT2, 47 wheat genotypes with the known *Pm* gene(s) were used as controls, including TAM104/Thatcher with *Pm*20 from 6RL, Kavkaz with *Pm8*, and Amigo with *Pm*17 that both located on 1RS, and CI14189 with *Pm7* from 2RL. Each genotype was tested with 22 single-pustule-derived powdery mildew isolates in the same way to compare their reaction patterns. A population of 97 F_2_ plants from a cross of YT2 and susceptible commercial cultivar Gao 8901 was used to determine the resistance source of powdery mildew resistance.

Wheat cultivars Chinese Spring (CS) and Holdfast, rye cultivars Weining and King II, 6R disomic addition line of Holdfast × King II (DA 6R), 6RS ditelosomic addition line (DtA 6RS), and 6RL ditelosomic addition line (DtA 6RL) were used to verify the specificity of molecular markers.

The complete set of disomic addition lines (DA 1R to 7R) of CS × Imperial, two hexaploid triticale lines 10R2-193-2 and 10R2-194-2 (AABBRR), and two T1BL⋅1RS wheat-rye translocation lines Lovrin 10 and Lovrin 13 (Rabinovich, 1998) were used as controls to characterize YT2 in molecular marker analysis.

### Sequential Genomic *in situ* Hybridization, Multicolor-Fluorescence *in situ* Hybridization, and Non-denaturing FISH Analyses

Genomic *in situ* hybridization and FISH were performed to determine the chromosomal composition of YT2. The mitotic metaphase chromosomes from root tips of YT2 were prepared and observed as previously described (An et al., 2013). Total genomic DNA of German White labeled with fluorescein-12-dUTP using nick translation method was used as a probe for GISH. Chromosomes were counterstained with 4′,6-diamidino-2-phenylindole (DAPI), and detection and visualization of rye chromatin were conducted according to An et al. (2013).

After rinsing the GISH hybridization probe signals, the probe pSc119.2 (green) labeled with fluorescein-12-dUTP combined with probe pAs1 (red) labeled with Texas-red-5-dCTP was used in mc-FISH detection. For ND-FISH detection, Oligo-pSc119.2-2 and Oligo-pTa535-2 were 5′-end-labeled with 6-carboxyfluorescein (6-FAM) producing green signals and 6-carboxytetramethylrhodamine (TAMRA) producing red signals, respectively. The FISH patterns were referred to Tang et al. (2014). These oligonucleotide probes were synthesized by Shanghai Invitrogen Biotechnology Co., Ltd. (Shanghai, China). Mc-FISH analysis was performed according to the method of An et al. (2013). ND-FISH analysis was operated according to Fu et al. (2015).

### Multicolor-GISH Analysis

Total genomic DNA was isolated from the fresh leaves of *Triticum urartu* (2n = 2x = 14, AA), *Aegilops speltoides* (2n = 2x = 14, BB), and *Aegilops tauschii* (2n = 2x = 14, DD). The total genomic DNA of rye and *T*. *urartu* was labeled with fluorescein-12-dUTP, and the total genomic DNA of *Ae*. *tauschii* was labeled with Texas-red-5-dCTP, while total genomic DNA of *Ae. speltoides* was used for blocking (Fu et al., 2012). Detection and visualization of mc-GISH signals were performed as the same as for GISH.

### Polymerase Chain Reaction Analysis

Three specific markers SW22057 (F: 5′-GAAGAGGACC GATGCCACTA-3′, R: 5′-TCACACTCCGGACAATGCTA-3′), SW22063 (F: 5′-TCTGCTTGATGATGATCTGCTT, R: 5′-TC CGCAAACCCTAACATTTC-3′), and SW5284 (F: 5′- CCTATC CCTCTGTGGCGAAT -3′, R: 5′- GTCGTGTTCCCCTACA TCCA -3′) were used to detect rye chromosome arms 6RS, 6RL, and 1RS, respectively (Han et al., 2020a). The marker O11B3/O11B5 (F: 5′-GGTACCAACAACAACAACCC-3′, R: 5′- GTTGCTGCTGAGGTTGGTTC-3′) was used as a diagnostic marker to detect wheat chromosome arm 1BS (Van Campenhout et al., 1995).

### Assessment of Resistance to Powdery Mildew

Seedling stage reactions of YT2 to 22 single-pustule-derived isolates were separately assessed with three replicates at the greenhouse as described (Qie et al., 2019). These isolates were collected from different wheat production regions of China and were characterized by their individual identifying host (Supplementary Table 1). Mingxian 169 and a set of 47 wheat genotypes with documented *Pm* gene(s) were used as controls (Supplementary Table 1). For each line, 20 seeds were planted in 128-well rectangular trays (54 cm × 28 cm × 4 cm), with 5 seeds per well (3 cm × 3 cm × 4 cm). After 15 days, when sporulation was observed on the first leaf of Mingxian 169, the plants were scored using a 0–4 scale with infection types (ITs) 0–2 considered resistant, while ITs 3–4 considered susceptible (Si et al., 1992).

Adult plant reactions to powdery mildew were tested on YT2, Shixin 616, 09R2-100, TAM104/Thatcher, CI14189, Kavkaz, and Amigo in field conditions, using three replicates during the 2018–2020 growing seasons at Luancheng Agro-Ecological Experimental Station, Chinese Academy of Sciences, Shijiazhuang, China (37°53′15′′N, 114°40′47′′E). The wheat cultivar Mingxian 169 was planted as susceptible control and inoculum spreader. In late March, Mingxian 169 was artificially inoculated with a mixture of *Bgt* isolates which mainly comprised virulent isolates E09, E18, and E20 collected from northern China. In May, when the susceptible control Mingxian 169 showed severe disease symptoms, the disease reaction of the tested plants at the adult stage was assessed using a 0–9 scale, in which 0–4 were considered resistant and 5–9 were considered susceptible (Sheng and Duan, 1991). Each plant was assessed twice for confirmation.

### Genetic Analysis of the Resistance to Powdery Mildew

To determine the resistance source of YT2, an F_2_ population of 97 plants derived from YT2 × Gao 8901 was tested for powdery mildew resistance. YT2, Gao 8901, and Mingxian 169 were also included in the test. At the seeding stage, the F_2_ plants were inoculated with *Bgt* isolate E09, a prevalent isolate in northern China (Li et al., 2011), to evaluate their seedling reactions and then transplanted into the field to test the adult plant reactions. Both seedling and adult plant reactions of each plant were assessed twice for confirmation. Three molecular markers, 6RS-specific marker SW22057, 6RL-specific marker SW22063, and 1RS-specific marker SW5284, combined with GISH were used to detect the rye chromatin in YT2.

### Development of 6RL-Specific Molecular Markers

To localize the powdery mildew resistance gene in a certain chromosome region, PCR-based markers specific for the 6RL chromosome were developed, based on the different reference sequences between Chinese Spring (International Wheat Genome Sequencing Consortium et al., 2018) and Weining rye (Li et al., 2021). All the primers were synthesized by Shanghai Sangon Biotechnology Co., Ltd. (Shanghai, China).

### Chromosomal Localization of Powdery Mildew Resistance Gene of YT2

^60^Coγ-ray radiation was used to develop progenies of YT2 containing translocations or deletions with different sizes of segments of chromosome 6R. At the flowering stage, the pollens of YT2 plants were treated with ^60^Co radiation at a dosage of 18 Gy and dosage rate of 1 Gy/min and then crossed with Gao 8901. To physically map the powdery mildew resistance locus, the irradiated F_1_ plants and their selfing F_2_ progenies were subject to GISH and molecular analysis and then tested for resistance to powdery mildew at the seedling and adult plant stages.

### Evaluation of Agronomic Traits

YT2 and its octoploid triticale parent 09R2-100 and wheat parent Shixin 616 were planted in the field. An evaluation of agronomic traits was conducted from 2018 to 2020 with three replicates in a randomized complete block design. Sowing and assessment methods were performed as described by Han et al. (2020b). At physiological maturity, plant height (PH), spike length (SL), spike number per plant (SNPP), fertile spikelet number per spike (FSS), thousand-kernel weight (TKW), kernel number per spike (KNS), and grain yield per plant (GYPP) of 20 randomly selected plants in the middle of the two internal rows were assessed. The analysis of variance (ANOVA) and least significant difference (LSD) test were performed using software SPSS 22.0 (SPSS Inc., Chicago, IL, United States) to test the significance of differences between YT2 and its parents Shixin 616 and 09R2-100 for each agronomic trait.

## Results

### Genomic *in situ* Hybridization Analysis of YT2

**FIGURE 1:**
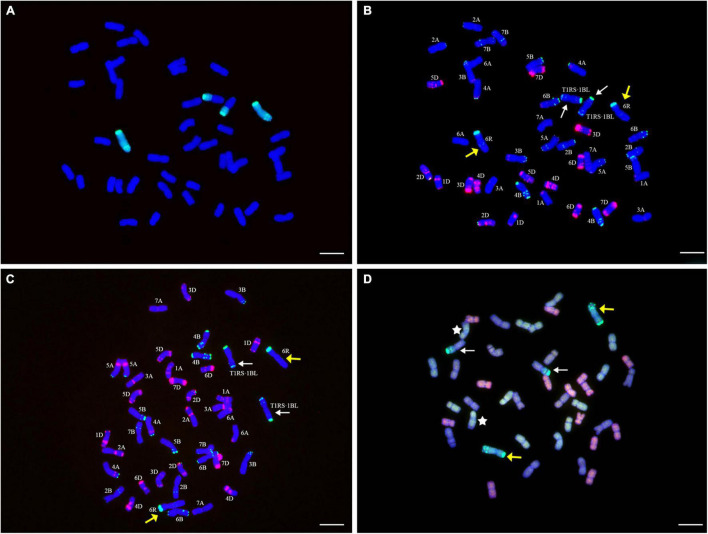
Genomic *in situ* hybridization (GISH), multicolor fluorescence *in situ* hybridization (mc-FISH), and multicolor-GISH (mc-GISH) identification of YT2. **(A)** GISH analysis of YT2 using total genomic DNA of rye cultivar German White (green) as a probe. **(B)** mc-FISH analysis of the same metaphase cell after GISH analysis **(A)** with pSc119.2 (green) and pAs1 (red). **(C)** ND-FISH analysis of YT2 with Oligo-pSc119.2-2 (green) and Oligo-pTa535-2 (red). **(D)** mc-GISH analysis of YT2, R-genome, A-genome, B-genome, and D-genome chromosomes displayed bright-green, yellow-green, brown, or gray, and pink or red fluorescence signals, respectively. The yellow arrows indicate the rye chromosomes 6R, the white arrows indicate the translocation chromosomes T1RS⋅1BL **(B–D)**, the white stars represent the T7A⋅4B translocations **(D)**, and the bar represents 10 μm.

The GISH results demonstrated that YT2 included 44 chromosomes in a cell and had one pair of intact chromosomes and two chromosome arms that displayed bright-green hybridization signals. The two chromosome arms contained an obvious secondary constriction and were translocated onto one pair of wheat chromosomes. The other chromosomes showed only the blue signals from counterstaining with DAPI (Figure 1A). The consistent results of GISH analysis for three consecutive generations of selfed YT2 further confirmed that it had high cytological stability.

### Fluorescence *in situ* Hybridization Analysis of YT2

After GISH analysis, mc-FISH, using the combination of probes pAs1 and pSc119.2, and ND-FISH, using the combination of probes Oligo-pSc119.2-2 and Oligo-pTa535-2, determined the identity of the individual chromosomes of YT2. The probe pSc119.2 produced strong green hybridization signals in the telomere regions of the short arm and four weak green points in the long arm of the intact chromosome. Meanwhile, strong green hybridization signals in the telomere regions and weak hybridization signals in the subtelomere regions on both the long and short arms of the translocated chromosomes were observed. The two introgressed rye chromosomes and chromosome arms did not bear any hybridization signals of pAs1 (Figure 1B). Using Oligo-pSc119.2-2 and Oligo-pTa535-2 as probes, the detection results were consistent with those by using pSc119.2 and pAs1 as probes (Figure 1C). Thus, the additional rye chromosomes were identified as 6R and the rye chromosome arm 1RS that translocated onto the wheat chromosome arm 1BL.

### Multicolor-GISH Analysis of YT2

To further reveal the genomic characteristics of YT2, mc-GISH analysis was performed by using genomic DNA of German White and three diploid progenitors of common wheat as probes or blockers. The results of the cross-hybridization signals made the rye chromosomes display green hybridization signals, whereas the wheat A-, B-, and D-genome chromosomes displayed yellow-green, brown or gray, and pink or red fluorescent signals, respectively (Figure 1D). The results demonstrated that YT2 had seven pairs of genome chromosomes for the A- and D-genome each, six pairs of B-genome chromosomes, one pair of rye chromosomes, and one pair of chromosome arms translocated onto one pair of B-genome chromosomes. The universal T7B⋅4A translocation in the wheat background was also observed in YT2 (Figure 1D).

### Polymerase Chain Reaction Analysis of YT2

**FIGURE 2:**
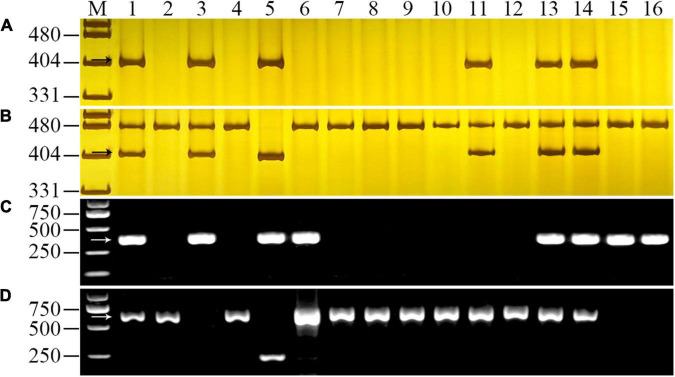
PCR amplification of 6RS-specific marker SW22057 **(A)**, 6RL-specific marker SW22063 **(B)**, 1RS-specific marker SW5284 **(C)**, and 1BS-diagnosed marker O11B3/O11B5 **(D)** for chromosome composition analysis of YT2. Lanes M, pUC19 *Msp*I in **(A,B)** and Trans2K Plus in **(C,D)**. 1, octoploid triticale 09R2-100; 2, Shixin 616; 3, YT2; 4, Chinese Spring; 5, Imperial rye; 6–12, 1R-7R disomic addition lines of “Chinese Spring × Imperial”; 13–14, two hexaploid triticale lines 10R2-193-2 and 10R2-194-2; 15–16, two T1BL⋅1RS translocation lines Lovrin 10 and Lovrin 13. The arrows indicated the diagnostic fragments of the markers.

Four molecular markers were used to confirm the chromosome composition of YT2. The 6RS-specific marker SW22057, 6RL-specific marker SW22063, and 1RS-specific marker SW5284 could amplify their approximate 400 bp targeted fragments in triticale parent 09R2-100 but not in wheat parent Shixin 616. By using markers SW22057 and SW22063, the corresponding DNA fragments were detected in YT2, DA 6R of CS × Imperial, two hexaploid triticale lines 10R2-193-2 and 10R2-194-2, but not in the other addition lines of CS × Imperial and T1BL⋅1RS translocation lines Lovrin 10 and Lovrin 13 (Figures 2A,B). In addition, YT2, 10R2-193-2, 10R2-194-2, Lovrin 10, and Lovrin 13 amplified the diagnostic fragments by using the 1RS-specific marker SW5284, but not in other corresponding genotypes (Figure 2C). Meanwhile, the 1BS-diagnostic marker O11B3/O11B5 amplified approximate 630 bp targeted fragments in all the tested genotypes except for YT2, Lovrin 10, and Lovrin 13 (Figure 2D). Therefore, YT2 was confirmed to bear chromosome 6R and chromosome arm 1RS, but not 1BS. Taking the results from the molecular marker and GISH/FISH analyses together, YT2 is a 6R disomic addition and T1RS⋅1BL translocation line.

### Responses of YT2 to Powdery Mildew

To further determine the resistance to powdery mildew, spectrum analysis to 22 *Bgt* isolates at the seedling stage was conducted on YT2 along with 47 wheat genotypes. YT2 was resistant to 12 of 22 *Bgt* isolates tested, showing a significantly different response spectrum in comparison with all the genotypes (Supplementary Table 1). Especially among the four genotypes with known *Pm* gene(s) from rye, TAM104/Thatcher with *Pm20* from 6RL of Prolific rye was resistant to 15 but susceptible to seven *Bgt* isolates. CI14189 with *Pm7* derived from 2RL of Rosen rye was highly susceptible to all the 22 isolates; Kavkaz with *Pm8* from 1RS of Petkus rye was resistant to isolates E50 and E60; Amigo with *Pm17* from 1RS of Insave rye was resistant to E06 and E13. In addition, compared with WR49-1, a wheat-rye 6R disomic addition line derived from wheat cultivar Xiaoyan 6 and rye cultivar German white that was previously identified by our laboratory, YT2 had a narrower response spectrum but was resistant to the four *Bgt* isolates E18, E19, E20, and E49, while WR49-1 was susceptible (An et al., 2015). These results suggest that YT2 may carry a new resistance gene.

During the 2018–2020 wheat-growing seasons, YT2, 09R2-100, and Shixin 616, along with TAM104/Thatcher, Kavkaz, CI14189, and Amigo, were inoculated with the mixture of *Bgt* isolates including E09, E18, and E20 collected from northern China. The results demonstrated that at the adult stage, YT2, 09R2-100, and TAM104/Thatcher were highly resistant that developed no mildew symptoms with IT 0, while Shixin 616 and the four controls Mingxian 169, Kavkaz, CI14189, and Amigo all showed susceptible reactions with ITs 8–9.

### Powdery Mildew Resistance Gene Is Conferred by Chromosome 6R

**FIGURE 3:**
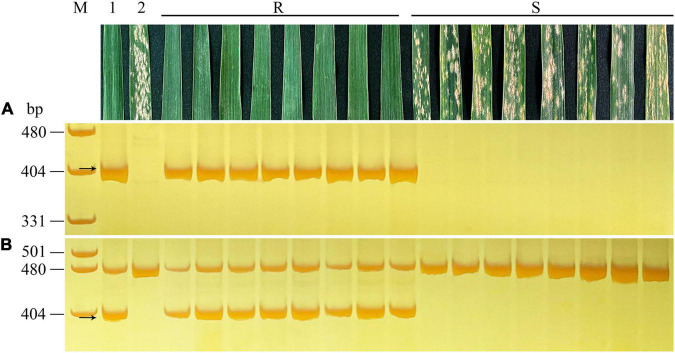
Evaluation of powdery mildew resistance at the adult plant stage and molecular detection of the 6R chromosomes from YT2 using 6RS-specific marker SW22057 **(A)** and 6RL-specific marker SW22063 **(B)** in F_2_ plants of YT2 × Gao 8901. Lanes M, pUC19 *Msp*I. 1, YT2; 2, Gao 8901; and R, resistant; S, susceptible F_2_ plants. The arrows indicate the diagnosed amplified fragments.

The F_2_ population of YT2 × Gao 8901 tested the reactions to powdery mildew resistance at both seedling and adult plant stages. All of the 97 F_2_ plants resistant or susceptible to *Bgt* isolate E09 at the seeding stage were identified by specific markers and GISH analysis for the detection of chromosomes 6R and 1RS. The result showed that all of the 50 resistant plants possessed at least one intact rye chromosome 6R, whereas the other 47 susceptible plants lacked any rye chromosome or only possessed the rye chromosome arm 1RS. At the adult plant stage, the F_2_ plants showed a consistent disease reaction that agreed with that of the seedling resistance test. Thus, co-segregation of the resistance phenotype with chromosome 6R indicated that powdery mildew resistance of YT2 was conferred by rye chromosome 6R (Figure 3).

### Development and Validation of 6RL-Specific Markers

**FIGURE 4:**
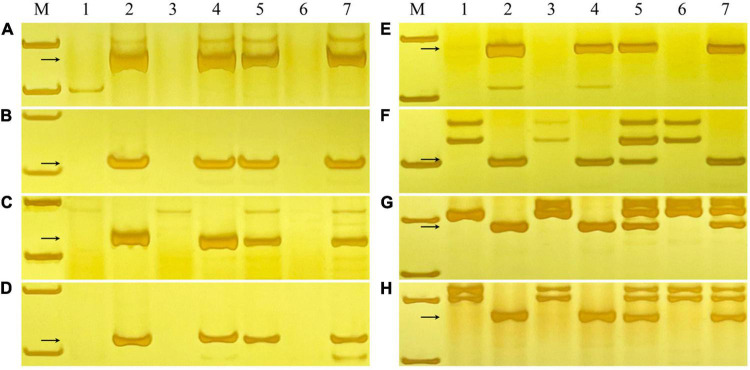
PCR amplification patterns of 6RL-specific markers *XM84*, *XM86*, *XM103*, *XM104*, *XM108*, *XMM20*, *XM127*, and *XMM47* **(A–H)**. Lanes M, pUC19 *Msp*I. 1, Chinese Spring; 2, Weining rye; 3, Holdfast; 4, King II rye; 5, DA 6R of Holdfast × King II; 6, DtA 6RS of Holdfast × King II; 7, DtA 6RL of Holdfast × King II. The arrows indicate the diagnosed amplified fragments.

A series of 6RL-specific markers with approximate 10 Mb apart were designed by comparing the sequences of the 6R chromosome of the Weining rye and CS genome. CS, Weining, Holdfast, King II, and DA 6R, DtA 6RS, DtA 6RL of Holdfast × King II were used to validate the specificity of the markers. For example, the newly developed markers *XM84*, *XM86*, *XM103*, *XM104*, *XM108*, *XMM20*, *XM127*, and *XMM47* were designed based on the locations of approximate 381.87 Mb, 390.02 Mb, 470.03 Mb, 479.87 Mb, 500.34 Mb, 581.48 Mb, 590.06 Mb, and 1023.93 Mb, respectively, corresponding to the 6R chromosome of Weining reference genome. All of these markers can only amplify the diagnostic fragments in Weining, King II, DA 6R, and DtA 6RL, but not in the CS, Holdfast, and DtA 6RS (Figure 4). From the centromere region to the terminal region of the 6RL chromosome, a total of 73 pairs of specific markers with certain locations were finally developed (Supplementary Table 2).

### Physical Localization of the Powdery Mildew Resistance Gene on Chromosome 6R

**FIGURE 5:**
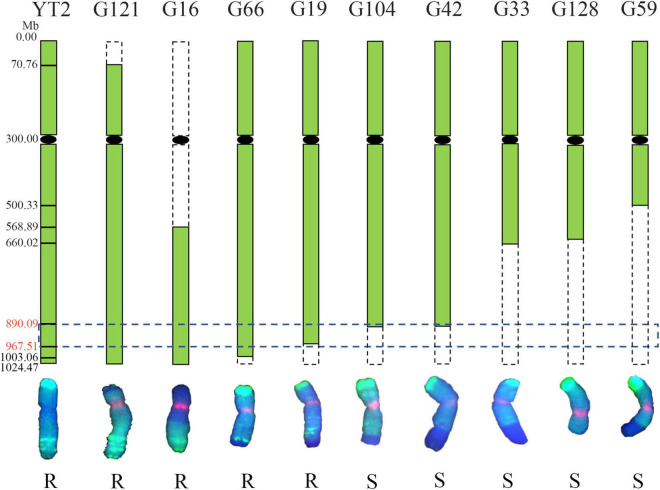
Physical mapping of the powdery mildew resistance gene of YT2. The green boxes represent 6R chromosomes, and the horizontal dotted box indicates that the powdery mildew resistance gene of YT2 was mapped to chromosome 6RL: 890.09–967.51 Mb, corresponding to the reference genome of Weining rye. The vertical dotted boxes represent missing chromosome segments of 6R. The bottom chromosomes were captured in GISH analysis and counterstained with 4′,6-diamidino-2-phenylindole (DAPI) (blue), and rye chromatin displays green signals. The letters R and S indicate the derivative lines that were resistant and susceptible to powdery mildew.

To confirm the physical location of the powdery mildew resistance gene on chromosome 6R, the irradiated F_1_ progenies were identified with GISH analysis and assessed for their resistance to powdery mildew at the seedling stage. As shown in Figure 5, nine variants containing different chromosome 6R segments were obtained. The results showed that translocation line G121 possessed intact 6RL chromosomes and was highly resistant to *Bgt* isolate E09, indicating that the powdery mildew resistance of YT2 was derived from 6RL. Translocation lines G16, G66, and G19 were resistant, while G104, G42, G59, and the deletion line G128 were susceptible. The chromosomal breakpoint positions of these variants were further determined using the 26 of 73 6RL-specific markers newly developed in this study. Combined with their GISH detection, responses to powdery mildew, and molecular marker analyses, the powdery mildew resistance gene of YT2 is mapped to the region in chromosome 6RL: 890.09–967.51 Mb, flanked by markers *XM189* and *X4M19*, corresponding to the reference genome of Weining rye. Disease responses and molecular marker analysis on their selfing F_2_ progenies also confirmed this result.

### Agronomic Performance of YT2

After strict bagging self-pollinating for five consecutive generations, neither morphology nor cytology segregation was observed on YT2. Compared to its parents 09R2-100 and Shixin 616, YT2 had a comparable performance on SNPP and KNS, but TKW and GYPP were significantly lower (Table 1).

**TABLE 1:** Agronomic traits of YT2, and its wheat parents Shixin 616 and triticale parent 09R2-100.

Year	Line	Plant height (cm)	Spike length (cm)	Spike number per plant	Fertile spikelet number per spike	Thousand kernel weight (g)	Kernel number per spike	Grain yield per plant (g)
2019–2020	YT2	77.3 ± 3.1^b^	7.2 ± 0.1^c^	13.7 ± 0.6^a^	18.0 ± 1.0^b^	26.0 ± 0.7^b^	51.0 ± 3.6^b^	12.2 ± 1.2^c^
	Shixin 616	67.7 ± 3.1^c^	9.4 ± 0.1^b^	13.7 ± 2.1^a^	19.3 ± 2.1^b^	40.0 ± 5.0^a^	59.3 ± 0.6^a^	18.8 ± 1.9^b^
	09R2-100	113.3 ± 6.4^a^	13.5 ± 0.1^a^	10.3 ± 0.6^b^	22.7 ± 0.6^a^	41.5 ± 2.1^a^	52.7 ± 1.5^b^	29.3 ± 1.7^a^
2018–2019	YT2	80.3 ± 2.4^b^	7.8 ± 0.5^b^	13.8 ± 1.5^a^	20.8 ± 0.5^a^	24.5 ± 1.1^c^	58.5 ± 3.5^a^	12.5 ± 1.8^c^
	Shixin 616	65.0 ± 1.4^c^	8.3 ± 0.5^b^	16.3 ± 1.7^a^	18.5 ± 1.3^b^	34.1 ± 2.5^b^	58.8 ± 5.5^a^	17.9 ± 2.3^b^
	09R2-100	91.8 ± 5.9^a^	13.4 ± 1.0^a^	10.3 ± 1.5^b^	22.0 ± 1.2^a^	40.1 ± 3.2^a^	52.3 ± 4.5^a^	27.2 ± 2.0^a^

*Values with the same letter in the same column were not significantly different at the P < 0.05 according to the least significant difference test.*

## Discussion

Rye, a naturally cross-pollinating relative of common wheat, which contains extensive genetic variation within and among cultivars, is an important gene donor for wheat improvement (Chikmawati et al., 2012; Targońska et al., 2016). Through distant hybridization and chromosome engineering, many wheat-rye addition, substitution, and translocation lines have been constantly developed as bridging parents in wheat chromosome engineering breeding (An et al., 2006, 2013, 2015, 2019, 2021; Han et al., 2022). In this study, a wheat-rye line YT2 was artificially selected from consecutive self-generations derived from the cross of octoploid triticale 09R2-100 and wheat cultivar Shixin 616, which exhibited high resistance to powdery mildew at both seedling and adult plant stages. Molecular cytogenetic identification, including GISH, mc-FISH, and specific molecular marker analysis, demonstrated that YT2 was a wheat-rye 6R disomic addition and T1RS⋅1BL translocation line. Genetic analysis indicated that the powdery mildew resistance was conferred by chromosome 6R. At the seedling stage, YT2 was resistant to 12 of 22 *Bgt* isolates, while at the adult plant stage, it exhibited high levels of resistance to a mixture of *Bgt* isolates collected in northern China, showing a significantly different response spectrum from the cataloged *Pm20*, *Pm7*, *Pm8*, and *Pm17* that also derived from rye. To physically map the resistance locus, a total of 73 markers specific for 6RL were developed and the resistance gene was finally localized to the region in chromosome 6RL: 890.09–967.51 Mb, suggesting that YT2 may carry a new powdery mildew resistance gene that differs from previously reported genes from rye.

YT2 was resistant to 12 of the 22 *Bgt* isolates tested at the seedling stage through resistance spectrum analysis, and it showed immunity to all the mixed *Bgt* isolates at the adult plant stage in the field. Some powdery mildew resistance genes have been reported from chromosome 6RL of rye cultivars including Prolific, Jingzhouheimai, and Kustro, among which the resistance gene from Kustro was localized in the region from the site between 2.3 and 2.5 to the telomere (Friebe et al., 1994; Wang et al., 2010; Li et al., 2016; Du et al., 2018). A powdery mildew APR gene(s) from the wild species *Secale africanum* was mapped on the long arm of 6R*^afr^* at FL0.85–1.00 (Li G.R. et al., 2020). Our laboratory also reported a wheat-rye 6R addition line carrying powdery mildew resistance gene on its 6R, while it showed significantly different reaction patterns in comparison with YT2 (An et al., 2015). Rye has strong heterogeneity, and many studies have shown different disease reactions of the derivatives even from the same original rye donor (Ren et al., 2011; Li et al., 2016; Schneider et al., 2016). Thus, the powdery mildew resistance gene in YT2 might be different from the previously reported genes from rye and could enrich the gene resources for wheat powdery mildew resistance breeding. Of course, fine mapping and cloning of the key genes should really explain their differences and the evolutionary relationship among those resistance stocks.

The realization of alien gene transfer plays an important role in improving the genetic diversity for wheat improvement (Zhang et al., 2016). Due to the extremely low recombination rate between alien chromosomes and wheat homoeologous counterparts, creating wheat-alien chromosomal translocations lines and deletion lines is one of the main means to determine the physical location of resistance genes derived from wheat relatives (Qi et al., 1998; Lukaszewski et al., 2001; Pu et al., 2015; Song et al., 2016; Zhang et al., 2016; Duan et al., 2017; Liu et al., 2017; He et al., 2018; Li J.B. et al., 2020). Given less linkage drag and regular meiotic behavior, small segment translocation lines are more preferred in wheat breeding practice (Falke et al., 2009; Qi et al., 2016; Ma et al., 2018). Therefore, as increasing resistance genes on rye chromosome 6R are identified, specific markers of high density on 6R appear of particular importance. They can rapidly anchor the targeted resistance regions based on the different phenotypes of different deletions and translocations, thereby promoting fine mapping and utilization of genes. In this study, according to the high-quality reference genome sequence of Weining rye (Li et al., 2021), a total of 73 6RL-specific PCR-based markers with an average density of approximate 10 Mb were developed. Using these developed markers and the deletion and translocation lines created by radiation, the powdery mildew resistance gene of YT2 was mapped to a certain region of the chromosome which is referred to as the reference genome of Weining rye. Based on these results, the creation of smaller segment translocations and the enrichment of interval markers will be more purposeful.

Whether a valuable disease resistance gene could be efficiently applied in resistance breeding largely depends on the agronomic traits of its carrier, but often, disease resistance is at the expense of some agronomic traits and reduced plant adaptation (Deng et al., 2017; Ma et al., 2018; Jia et al., 2020). Compared with the previously reported chromosome addition lines such as WR49-1, YT2 carried an additional T1RS⋅1BL chromosome translocation, which may have an additional positive value on breeding utilization of YT2. Among all of the rye translocation lines, 1RS translocation line was regarded as particularly notable success in wheat breeding because it carried multiple resistance genes against powdery mildew, stripe rust, leaf rust, and stem rust, as well as genes associated with superior agronomic traits and abiotic-stress tolerance (Lukaszewski, 1990; Kumlay et al., 2003; Shearman et al., 2005; Howell et al., 2014, 2019). In spite of the newly variant pathogen virulence circumventing the disease resistance, the T1RS⋅1BL translocations have continued to be widely utilized in wheat breeding because of their yield potential and wide environmental adaptability (Ren et al., 2017; Wang et al., 2017). In this study, the 1RS of YT2 inherited from its triticale parent showed no resistance to powdery mildew, but its role in agronomic traits should not be underestimated in future work. In addition, it is notable that T1RS⋅1BL translocations increased yield in only specific combinations of 1RS and wheat genetic background (Jung and Lelley, 1985; Ren et al., 2017). To clarify the future breeding strategy of YT2, comprehensive evaluations of agronomic traits were conducted on YT2 and their parents 09R2-100 and Shixin 616. Compared to its wheat parent Shixin 616, YT2 had significantly lower TKW and GYYP, probably due to its small and shriveled seeds. Thus, YT2 could be designed to cross with various wheat cultivars with good performance on TKW and GYPP while transferring its powdery mildew resistance.

## Conclusion

In this study, we developed and identified a novel wheat-rye 6R addition and T1RS⋅1BL translocation line YT2 with high powdery mildew resistance, developed new rye chromosome-specific markers, and further mapped its powdery mildew resistance locus. This study provides an efficient model from developing germplasm to comprehensively and accurately identifying it. The new germplasm YT2 can serve as a promising genetic stock in wheat improvement.

## Data Availability Statement

The datasets presented in this study can be found in online repositories. The names of the repository/repositories and accession number(s) can be found in the article/Supplementary Material.

## Author Contributions

DA, GH, and HY conceived the research. GH, HY, JW, LC, and SL performed the experiments. LL and XL contributed to the development of the materials. YZ and JF performed phenotypic assessments. GH and HY wrote the manuscript. DA and LL supervised the research. All authors read and approved the final manuscript.

## Conflict of Interest

The authors declare that the research was conducted in the absence of any commercial or financial relationships that could be construed as a potential conflict of interest.

## Publisher’s Note

All claims expressed in this article are solely those of the authors and do not necessarily represent those of their affiliated organizations, or those of the publisher, the editors and the reviewers. Any product that may be evaluated in this article, or claim that may be made by its manufacturer, is not guaranteed or endorsed by the publisher.
